# Features of Microstructure and Texture Formation of Large-Sized Blocks of C11000 Copper Produced by Electron Beam Wire-Feed Additive Technology

**DOI:** 10.3390/ma15030814

**Published:** 2022-01-21

**Authors:** Kseniya Osipovich, Andrey Vorontsov, Andrey Chumaevskii, Evgeny Moskvichev, Ivan Zakharevich, Artem Dobrovolsky, Alexander Sudarikov, Anna Zykova, Valery Rubtsov, Evgeny Kolubaev

**Affiliations:** Institute of Strength Physics and Materials Science, Siberian Branch of Russian Academy of Sciences, 634055 Tomsk, Russia; vav@ispms.ru (A.V.); tch7av@gmail.com (A.C.); em_tsu@mail.ru (E.M.); ivan.zaharevich@yandex.ru (I.Z.); artdobrov@ispms.ru (A.D.); avsudarikov@ispms.ru (A.S.); zykovaap@mail.ru (A.Z.); rvy@ispms.ru (V.R.); eak@ispms.ru (E.K.)

**Keywords:** additive manufacturing, electron beam wire 3D printing, copper alloys, large-sized blocks, microstructure, texture

## Abstract

The paper investigated the possibility of obtaining large-sized blocks of C11000 copper on stainless steel substrates via electron beam wire-feed additive technology. The features of the microstructure and grain texture formation and their influence on the mechanical properties and anisotropy were revealed. A strategy of printing large-sized C11000 copper was determined, which consists of perimeter formation followed by the filling of the internal layer volume. This allows us to avoid the formation of defects in the form of drops, underflows and macrogeometry disturbances. It was found that the deposition of the first layers of C11000 copper on a steel substrate results in rapid heat dissipation and the diffusion of steel components (Fe, Cr and Ni) into the C11000 layers, which promotes the formation of equiaxed grains of size 8.94 ± 0.04 μm. As the blocks grow, directional grain growth occurs close to the <101> orientation, whose size reaches 1086.45 ± 57.13 μm. It is shown that the additive growing of large-sized C11000 copper leads to the anisotropy of mechanical properties due to non-uniform grain structure. The tensile strength in the opposite growing direction near the substrate is 394 ± 10 MPa and decreases to 249 ± 10 MPa as the C11000 blocks grows. In the growing direction, the tensile strength is 145 ± 10 MPa.

## 1. Introduction

Additive manufacturing (AM) covers an increasing range of tasks in industry. Currently, common additive manufacturing technologies are electron beam melting (EBM) [1], selective laser melting (SLM) [2], direct energy deposition (DED) [3], wire and arc additive manufacturing (WAAM) [4] and electron beam additive manufacturing (EBAM) [5]. The first two technologies refer to the powder types of AM, and they have advantages related to the production of multicomponent systems, such as high-entropy alloys [6]. Therefore, the most productive AM technologies are considered to be DED, WAAM and EBAM. EBAM is based on melting a metal wire with an electron beam in a vacuum. The product is printed layer by layer until it is fully formed. EBAM technology is effective for the production of metal parts with complex geometries and low material costs [7]. The positive aspects of the technology include printing directly in a vacuum chamber, which makes it possible to work with materials that are sensitive to oxidation. Electron beam printing is suitable for most materials, including the laser radiation of highly reflective alloys, such as Al and Cu [8]. 

Cu is one of the most widely used constructional materials due to its excellent electrical conductivity, thermal conductivity, ductility, machinability and formability. Due to this, Cu has a wide range of practical applications, and it is used in various engineering industries, such as in the automotive, aerospace, electrical power, electronics and defense industries, etc. The high thermal conductivity of copper and copper alloys allows for the manufacture of various components, such as heat exchangers (combustion chambers for liquid rocket engines), power units, springs and bearings and electronic connectors in the above sectors. These high heat flux environments require a high-strength, high-conductivity alloy to properly cool the thrust chamber walls with high-pressure rocket fuel or an oxidizer. The higher thermal conductivity of pure copper leads to faster heat dissipation and a higher localized temperature gradient, which causes the twisting and delamination of the deposited layer [9]. The high thermal conductivity of copper quickly dissipates heat away from the molten pool, causing temperature gradients and distortions. This is complicated by a significant difference in the thermophysical properties of the compacted material and the surrounding powder layer. Superior electrical conductivity means that copper alloys are widely used in the manufacture of thermal transformers, radiators, combustion chambers and other components because of their superior electrical and thermal conductivity [10,11,12,13]. The higher reflectivity of pure copper reduces the amount of energy available for melting, and makes it detrimental to laser optics. The tendency of Cu to form oxides during processing leads to the agglomeration and reduced fluidity of molten copper, resulting in the reduced wettability of pure Cu due to copper oxide formation [14,15]. Copper is used in many applications due to its high thermal conductivity (401 W/(m∙grad)) and electrical conductivity (58 MSm/m). In addition, copper has high corrosion resistance, good machinability and a low enough price, which makes it widely used in electrical and mechanical engineering industries [16]. Air tuyeres are one of the most important elements of blast furnace design that determine the efficiency of its operation. The tuyere is designed for blast furnace blowing with gas (air, oxygen or other) to ensure fuel combustion. In this case, additive technologies are effective in terms of material and energy consumption for tuyere production. In addition, high cooling rates can lead to small grain sizes in the structural parts, resulting in improved mechanical properties [17,18].

The microstructure during solidification in manufacturing is determined by the temperature gradient, solidification rate, cooling and other material-related parameters [19,20]. The small size of the melt pool, the strong recirculation motion of the liquid alloy caused mainly by Marangoni convection [21], heat source movement and rapid temperature changes make it difficult to accurately measure the temperature gradient and other important variables. Consequently, many AM processes lead to the formation of metastable microstructures and non-equilibrium compositions of the resulting phases, which may even vary for each layer of the deposited material [22,23]. This makes the modeling of microstructures and compositions in fabricated AM parts difficult and complicated. Obviously, the temperature gradient depends on a number of process parameters, such as energy density, layer thickness and preheating temperature [24,25]. In this regard, solidification occurs with the formation of a cellular dendritic structure [26]. Each new layer to be deposited partially remelts already solidified layers, which act as substrates [24,27,28]. Consequently, the new layer is formed according to the same crystalline orientation as the underlying, already solidified layers, although the growth direction may change. This principle is used to form the crystallographic texture or, in other words, epitaxial growth [26,27,29].

The grain structure and crystallographic texture of the component depend on the melting and crystallization of the melt pool. The shape of the melt pool in this case varies depending on the printing parameters, and can be oval or curved on the top surface and semicircular or keyhole-shaped in cross-section, depending on the intensity of the heat source and the rate of movement. Growth occurs during solidification because of previously deposited layers, and this determines the crystallographic essence of the structure through the partial or complete melt feedback of the previously formed layer. In addition, crystallographic texture is also determined by the scanning strategies used during sample fabrication. In the case of IN718, it was observed that the texture was designed with a unidirectional scanning strategy, whereas the rotated cube texture was designed with bidirectional scanning. These textures are a consequence of the compromise between the directions of easy grain growth and the directions of maximum heat flux near the boundaries of the melt pool [30]. Growth is preferable only when the direction of growth coincides with the direction of maximum heat flux. Such features of crystallization can be caused by the parameters of technological processes of manufacturing, and the heterogeneity of heat removal from the billet during additive growth. Typically, columnar grains normally grow along the boundary of the melt pool, where there is no significant supercooling. Columnar grains grow as the bottom of the melt pool and move upward during the subsequent cooling process. These grains grow continuously by shifting the direction of their growth according to the temperature gradient. Thus, macroscopically, it appears that the curved columnar grains "stretch" behind the curvature of the trailing edge of the melt pool.

However, little is known about the use of crystallographic texture and grain size as a tool for designing a functional microstructure, and even less is known about the relationship between the preferred anisotropy, microstructure and mechanical properties. Therefore, the goal of this work was to obtain large-sized workpieces to investigate texture formation and its effect on the anisotropy of mechanical properties under tension in different directions relative to growth.

## 2. Materials and Methods

**Figure 1:**
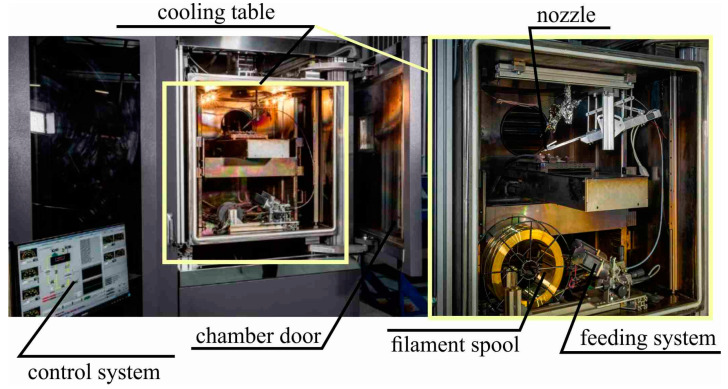
Additive electron beam printing setup with wire filament.

Large-sized samples of C11000 copper were obtained through electron beam wire-feed additive manufacturing with experimental equipment at the Institute of Strength Physics and Materials Science, Siberian Branch of the Russian Academy of Sciences (Tomsk, Russia) (Figure 1).

An AISI 304 stainless steel substrate and technical grade C11000 copper wire were used to obtain the samples (Table 1). The diameter of the copper wire was 1.6 mm.

**Figure 2:**
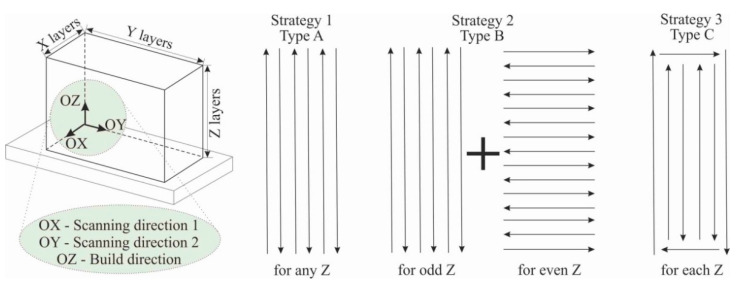
Schematics of scanning strategies for obtaining large-sized copper samples via electron beam wire-feed additive technology.

The steel substrate was fixed on a three-axis water-cooled table. An electron beam was used to form a melt pool. The wire was fed through a nozzle into the melt pool. The voltage during printing was constant at U = 30 kV. For the first layers of the large-sized copper samples, the current and printing speed were I_I_ = 90 A and υ_I_ = 300 mm/min, respectively. Further, as the height of the large samples increased, the current and printing speed were changed to I_II_ = 60 A and υ_II_ = 400 mm/min, respectively. The wire feed rate was 1.5–2.0 mm/min, depending on the printing speed. The width of the melt pool was 6 mm and the distance between passes was 5 mm. The formation of the melt pool was performed by sweeping an electron beam, in the form of a circle with a diameter of 5 mm, at a frequency of 1 kHz. The thickness of the deposited layer was 1 mm. Large copper blocks were obtained according to three types (A, B and C) of scanning strategy (Figure 2). Blocks of type A were obtained by printing with the same scanning direction for any layer along the Z axis, i.e., along the direction of large-sized sample growth. The OX and OY axes are related to scanning directions 1 and 2, respectively. Strategy 1 (type A) involves a line being interrupted by alternating forward and backward passes without changing the printing direction on each layer along the OX and OY axes. When printing blocks of type B, scanning strategy 2 was used, which involved keeping the pattern along the OY axis, as in strategy 1. However, on each even-numbered layer, a 90° clockwise rotation was performed along the growing direction. In type C blocks, perimeter formation was performed first, followed by filling the inner volume of the layer.

Thus, three main types of copper blocks of types A, B and C, differing according to the technological features of production, were achieved. The obtained volumetric samples had the form of parallelepipeds. Type A blocks were 150 mm long, 40 mm wide and 40 mm high. Type B blocks were 150 mm long and 40 mm wide. The final maximum height of the sample was not achieved due to the unsuitability of strategy 2, which led to the formation of defects in the workpiece. Type C blocks had a length of 150 mm, a width of 40 mm and a height of 80 mm.

**Figure 3:**
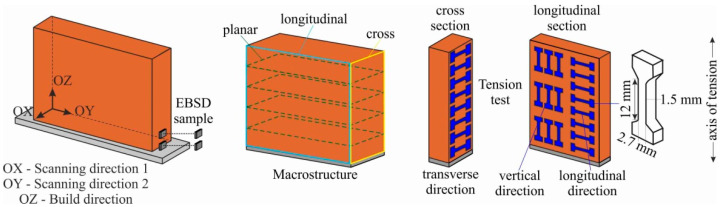
Scheme of cutting samples into metallographic sections and samples for mechanical testing.

From the thus-grown large-sized copper blocks, samples were cut using a DK7750 electrical discharge machine to study the macro- and microstructure. In order to study the structure and mechanical tensile tests, the samples were cut according to the scheme shown in Figure 3.

Before the structural studies, the samples were ground and polished with diamond paste followed by the etching of the polished surfaces of the samples in a solution of 10 mL HCl + 1 g FeCl_3_ + 20 mL H_2_O. Macrostructural studies were performed using an OLYMPUSLEXT confocal microscope (Olympus NDT, Inc., Waltham, MA, USA). To determine the elemental composition of the specimens, a Zeiss LEO EVO 50 (Carl Zeiss, Oberkochen, Germany) scanning electron microscope (SEM) was used. Microhardness was measured using a Duramin5 (Struers A/S, Ballerup, Denmark) machine. Microhardness measurements were carried out via the Vickers method at a load of 50 g for 10 s. The tensile specimens were in the shape of a double-sided blade. The working part of the double-sided blade was 12 mm (width: 2.7 mm, thickness: 1.5 mm). Tests were performed on a universal testing machine, UTS 110M (Testsystems, Ivanovo, Russia). The strain rate was 1.4 × 10^−3^ c^−1^. Macroscopic images of the specimens were taken with a Pentax K-3 camera with a lens focal length of 100 mm. Microstructural studies were performed using a scanning electron microscope, Tescan MIRA 3 LMU (TESCAN ORSAY HOLDING, Brno, Czech Republic), equipped with a system of registration of back-reflected electrons, Oxford Instruments Nordlys, and an energy dispersive X-ray spectrometer, Oxford Instruments Ultim Max 40 (Oxford Instruments, High Wycombe, UK). The scans were performed at an accelerating voltage (HV) of 20 kV and a beam current of 10 nA. Electron backscattered diffraction (EBSD) samples were prepared using the standard technique: sandpaper grinding to #2000, diamond polishing from 9 to 1 µm and 50 nm col-K suspension fine polishing. The final stage of sample preparation was ion polishing for 10 min at 10 kV by using a Technoorg Linda SEMPrep2 ion mill (Technoorg Linda Co. Ltd., Budapest, Hungary) with a wide plane-parallel argon ion beam. 

## 3. Results and Discussion

### 3.1. Choosing a Scanning Strategy for Large-Sized C11000 Blocks

**Figure 4:**
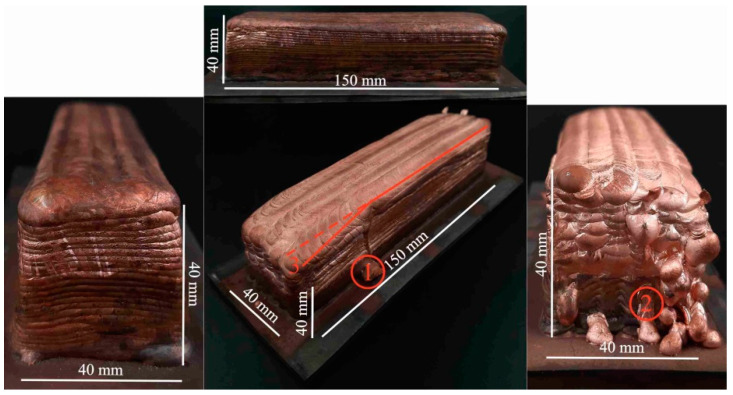
Macrostructure of A-type blocks fabricated using scanning strategy 2 with electron beam wire-feed additive technology. Noted defects by type: 1—droplets, 2—leaks and 3—decrease in the height.

**Figure 5:**
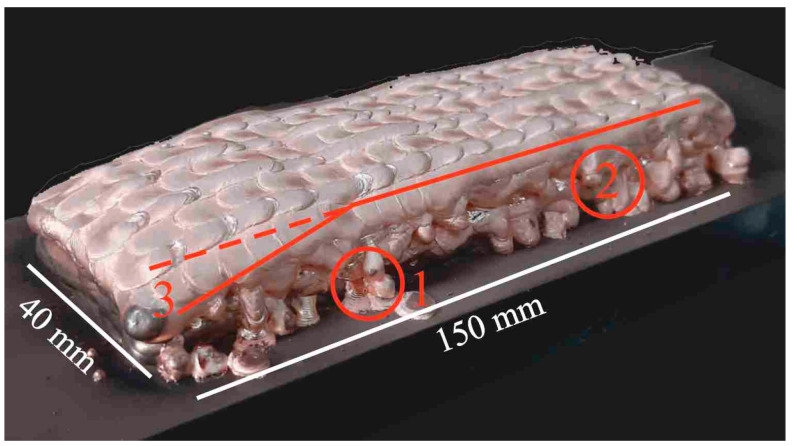
Macrostructure of B-type blocks fabricated using scanning strategy 2 with electron beam wire-feed additive technology. Noted defects by type: 1—droplets, 2—leaks and 3—decrease in the height.

**Figure 6:**
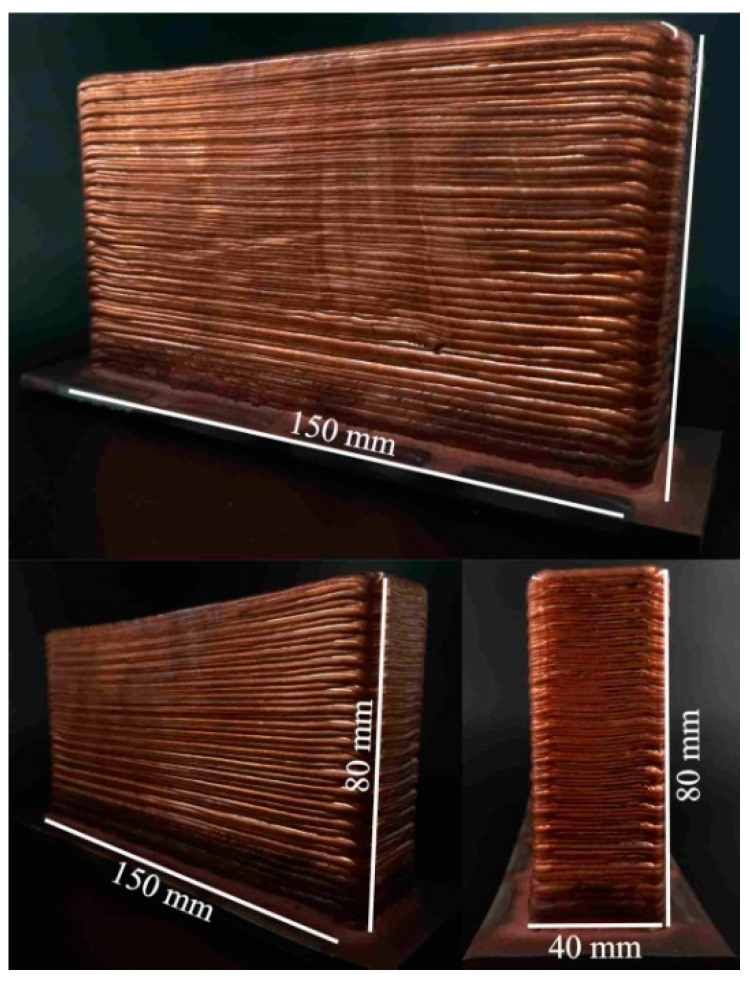
The macrostructure of C-type blocks fabricated using scanning strategy 3 with electron beam wire-feed additive technology.

**Figure 7:**
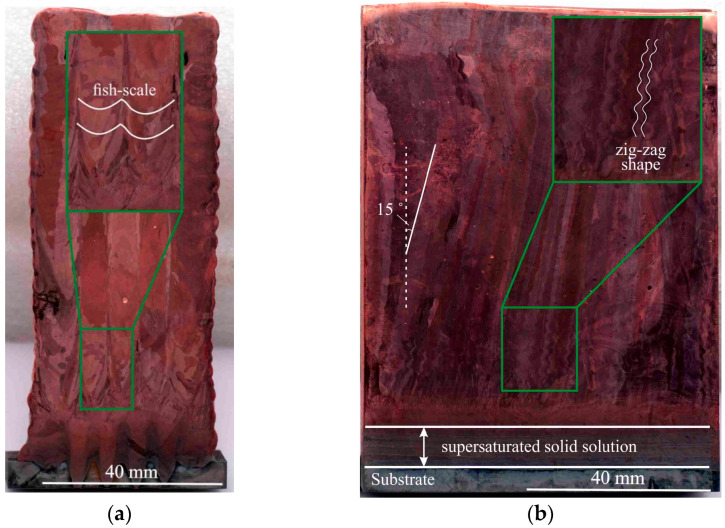
Macrostructure of the C-type copper block in cross- (**a**) and longitudinal sections (**b**). The green rectangle indicates the enlarged area showing the fish-scale and zig-zag shape of the structure.

**Figure 8:**
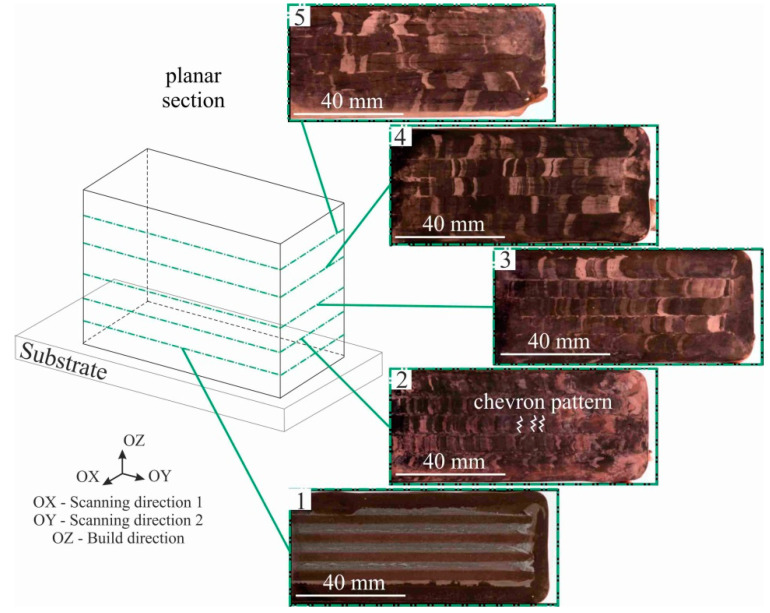
Macrostructure of the C-type copper block in planar section. The green dotted lines show areas of distance from the substrate—area 1: 1.5 mm, area 2: 3 mm, area 3: 4.5 mm, area 4: 6 mm, area 5: 7.5 mm. The white line shows the chevron pattern structure.

The general view of the large-sized copper blocks grown through electron beam wire-feed additive technology is shown in Figure 4, Figure 5, Figure 6, Figure 7 and Figure 8. For type A blocks grown using scanning strategy 1 (Figure 2), macrogeometric disruption is observed in the last layers. At the same time, a number of defects in the form of droplets, leaks and a decrease in the height of the blocks in the upper part (Figure 4) occurred. As the large-sized sample was grown, the substrate temperature increased locally near the melt pool. As a result, a temperature gradient occurred, which led to heat accumulation. After a number of thermal cycles, the deposited solidified layers were deformed under the influence of temperature stresses, which led to the formation of defects on the end part of the sample.

Printing type B blocks using strategy 2 (Figure 2) involved alternating the direction of layer deposition according to the scheme. However, there was a violation of the continuity of material flow on the surface of the workpiece, which led to the formation of drops and leaks of material. Strategy 2 implied minimizing the temperature gradient in all directions, primarily opposite to the orientation of the assembly. However, as a result, a large number of defects were formed, and the geometry of the samples was disrupted (Figure 5).

The correction of such defects was achieved by changing the printing scheme. In type C blocks, the perimeter was formed first, followed by the filling of the inner layer volume. The blocks obtained using this printing scheme did not show the presence of geometric distortions, even when reaching a height of 80 mm (Figure 6). The surface of such samples is quite evenly distributed, with parallel layers without bends in the layers or stacking irregularities, and the proportions of the shape are maintained. The formation of the perimeter creates conditions for high heat dissipation, affecting the water-cooled table when carrying the first layers and creating a radiation effect when depositing the last layers. This leads to the differences in the structure and properties of the sample described below.

Thus, for the production of blocks with correct geometry and compliance with the proportion of the shape of large-sized copper blocks using electron beam wire-feed additive technology, suitable scanning involves the initial formation of the perimeter and the subsequent filling of the inner layer volume (strategy 3, type C). Since the type C printing strategy proved to be preferable, in the following section, we will consider the structure and mechanical properties of the blocks obtained through strategy 3 only.

### 3.2. Macrostructure and Microhardness of Large-Sized C11000 Blocks

In the structure of C-type copper blocks, a coarse grain structure is formed with preferential growth in the direction of heat removal (Figure 7). Typical structures can be observed for additively manufactured workpieces, in which overlapping semi-elliptical melt pools are formed (Figure 7a). The formation of semi-elliptical melt pools is characteristic of various additive manufacturing methods, both for ferrous [31], as well as for non-ferrous metals [32], which is associated with the features of the process. The boundaries of the melt pool in the sample are visible as “fish-scales” [33,34]. The geometry of the melt pool is determined by the process parameters, material properties and cooling conditions. The cross-sectional structure of a large-sized copper sample is characterized by the evolution of the structure during sample fabrication. According to the macrostructure shown in Figure 7a, directional solidification occurs on the first layers near the substrate with fine grain sizes. Moreover, the grain on the first layers near the substrate is equiaxial. Directional solidification is accompanied by the growth of columnar grains that extend in the growing direction of the workpiece. Directional grain growth is facilitated by the slow heat dissipation and temperature gradient formed during additive growth of the workpiece. In the longitudinal section (Figure 7b), the macrostructural analysis reveals areas with banded copper grains. The gradual development of columnar grains may not always follow the normal path to the curvilinear surface, as defined by the liquidus isotherm. For the actual solidification, the surface deviates significantly from the liquidus isotherm due to the effects of supercooling. For example, one paper shows the effects of supercooling on grain growth directions [35], where there are noticeable discrepancies between the actual solidification surface and the liquidus isotherm in systems in which there is significant supercooling. It is possible to observe a noticeable deviation of the actual curing surface from the liquidus isotherm, which further increases with the printing speed. However, the degree of deviation, determined by the degree of supercooling, must be considered for specific solidification conditions. “Zigzag” grains can form at high cooling rates when the heat trace moves behind the melt pool. When the heat gradient aligns across the specimen, flatter grains form. The direction of melt pool formation in one layer is opposite to the direction in the other layer, causing the grain to grow obliquely along one of the directions perpendicular to the surface. Growth will spread to the next layer if the scanning direction remains unchanged for the next layer, but if the scanning direction is reversed, growth will be aligned further away from the direction of maximum heating. The scanning strategy is crucial for changing the temperature gradient and, consequently, the texture of the material [30]. In this work, large-sized samples were made of copper, which, having high thermal conductivity, effectively removes heat from the melt pool. These areas are located in the lower and upper parts of the workpiece (Figure 8). In the cross-section, the direction of grain growth is vertical, and in the longitudinal section, there is a 15˚ angle bend in the grains (Figure 7b). The tilt of the dendritic structure is related to the deviation of the direction of the temperature gradient during crystallization. The orientation of the grain structure depends on the shape and size of the melt pool. Grains growing at high printing speeds in this process are fairly straight. As a rule, during solidification, elongated grains grow from the edge to the center of the melt pool, depending on its location. The direction of heat flow in this case depends largely on the local positions at the melt pool boundary [16,30,36,37,38].

The analysis of the macrostructure in the longitudinal section (Figure 7b) shows that the sample has a clear interface. No crack-like defects are detected along the interface, indicating the complete deposition of the layers. There are no shrinkage pores, which are usually formed during the deposition of the first layers of the material, and during direct contact with the substrate. At the moment of wire-feed melting by the electron beam and the formation of the melt pool, there is a local increase in the substrate temperature near the melt pool. As a result, there is a temperature gradient, which leads to the presence of internal stresses. After a number of thermal cycles, the substrate deforms under the influence of the thermal stresses, and its significant intrusion occurs, but does not affect the quality of the large-sized copper blocks (Figure 7a). This is due to the peculiarity of sample fabrication, since AM implies a partial remelting of the previous layers. As a result of the mutual mixing of the Cu deposited in the first layers and the steel elements remelted from the substrate, the formation of the combined phases of the Cu-Fe system does not occur. A diffusion layer of supersaturated solid solution is formed at the concentration limit of these components, and elements such as solid solutions of copper in iron, and iron in copper with the additional mutual dissolution of the alloying elements, as well as mechanical mixtures of the system components, are formed in this region (Figure 7b). In the fusion region of the copper sample with the steel substrate, the content of steel particles in the copper gradually decreases, which can be clearly seen in the longitudinal section in Figure 8, area 1. 

The actual grain growth direction can differ significantly from the local maximum temperature gradient, depending on the scanning strategy. “Zigzag” patterns have major grain directions that are perpendicular to each other in successive layers, resulting in a 45° angle to the scanning direction. This results in epitaxial grain growth in the first layer shifting to grain formation in a different direction in the second layer, or vice versa, with a 15° deviation between the maximum heat flux temperatures. Thus, texture evolution is strongly influenced by the scanning strategies that correlate with the directions of easy growth and maximum heat flux. Full alignment results in a texture similar to the norm. The final solidification texture is determined by local heat flux directions and competitive grain growth.

The horizontal section is distinguished by the main structural components of the blocks (Figure 8). In the upper part of the large-sized sample, there are large, elongated grains, and in the lower part of the sample, there is an equiaxed fine-grained structure. 

**Figure 9:**
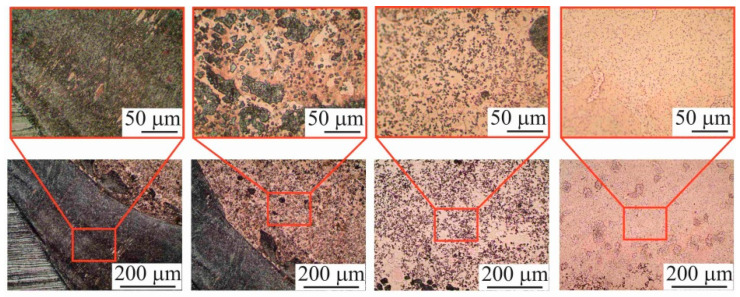
The microstructure of the C-type copper block in the transitional boundary.

In general, the structure of the sample is a columnar grain crossing most of the layers. In the lower part of the blocks, i.e., part 1 (Figure 8, area 1), there is an area that is affected by the substrate due to the intense intrusion of steel into the first layers of printed copper. The greatest substrate intrusion was characteristic of the central part of the blocks. At the same time, the structure of the blocks at the macrolevel is represented by a constant alternation of the columns printed according to the scheme shown in Figure 2. The surface of the large-sized sample in the longitudinal section demonstrates the presence of a chevron-like pattern [39] due to grain growth towards the moving heat source from the partially melted wire filament. Since the local thermal conductivity is affected by the scanning strategy, it was found that the orientation of the dendrites also depends on the scanning strategy. A chevron-like pattern perpendicular to the growth direction of the sample and the longitudinal direction of the layer deposition was found for the zigzag scanning pattern (Figure 8, part 2). The grains grow towards the melt pool when the scanning orientation is unidirectional. The structure at a higher height from the substrate (30 mm—area 3, 45 mm—area 4, 60 mm—area 5) presents a similar structure, but with a constant increase in the grain size (Figure 8, parts 3–5): at 30 mm from the substrate, the grain size is 2.7 mm, at 45 mm the grain size is 4.7 mm and at 60 mm the grain size is 7 mm. Besides, a gradual decrease in material etchability is observed, so that with the same etching conditions, the boundaries between the deposited layers on the background of the grain structure become clearly visible in the last layers. This is due to the transfer of material under the influence of the temperature gradient. Since the melt pool has an elliptical shape, the electron beam was concentrated in the central part of the beam sweep; therefore, the temperature in the central part of the melt pool was higher than at its edges. In the next part of the blocks, area 2, 15 mm above the substrate, the mixed steel was practically absent, but the structure was rather finely dispersed (Figure 8, part 2). The presence of impurity atoms (near the substrate) can be explained by the fact that the electron beam melts not only the previous layer, but also part of the underlying material, in this case, the substrate. Spherical copper inclusions are observed in this area (Figure 9), and it has increased etchability compared to the austenitic steel substrate. The analysis of the data in Figure 9 suggests that the austenitic steel is being alloyed with copper atoms near the substrate to form a two-phase region of copper and copper-alloyed austenitic stainless steel. This does not contradict the known data on the Fe-Cu phase diagram [40], according to which intermetallic compounds on the basis of iron and copper are not formed. However, the dissolution of a limited amount of copper (≈5.8% at 1083 °C) in γ-Fe is possible. As the number of spherical copper inclusions decreases with distance from the interface, the concentration of copper also becomes lower.

Near the interface in the copper part of the bimetal, we also observed inclusions with weak etching, and a morphology close to the structure of austenitic steel below the interface “substrate-additive layers” (Figure 9). The presence of a small concentration of iron in the copper part of the bimetal sample near the interface can be caused by the formation of a solid solution of iron in copper, although the solubility of iron in copper is quite low (≈2.8% at 1083 °C) [40]. Thus, the phase composition changes according to the following sequence:

γ-Fe (Cr, Ni, Cu) + ε-Cu,Fe → γ-Cu,Fe + γ-Fe (Cr, Ni, Cu) → ε-Cu + ε-Cu,Fe → ε-Cu.(1)

At a distance of approximately 1.5 mm from the interface, the Cu content is 98.9 (wt. %), which is close to the grade composition of C11000 copper. A clear transition zone, indicating the formation of a solid solution of iron in copper, on the metallographic thin sections is not revealed.

**Figure 10:**
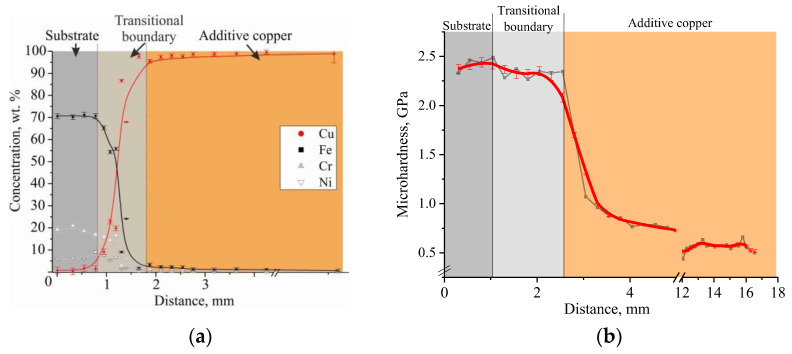
Changes in the elemental composition wt. % (SEM EDS analysis) (**a**) and dependence of the microhardness values (**b**) with the distance from the substrate of C-type copper block.

Thus, the first layers have a high concentration of substrate chemical elements, such as Fe, Ni and Cr, whose concentration decreases as the C11000 blocks is printed (Figure 10a).

The analysis of the distribution of microhardness at transition through an interface of a bimetallic sample testifies to the sharp change in the strength properties in a material, which goes from 2.29 GPa, corresponding to austenitic steel, to 0.54 GPa in the copper part of a sample. The transition zones near the interface described above are characterized by scattered microhardness values (in the steel part of the bimetal) and elevated microhardness values (in the copper part of the bimetal) (Figure 10b). The variation in the steel part of the sample near the interface is due to the fact that, during the microhardness measurement, the indentor is partially caught by the “soft” copper inclusions in the transition zone. In the copper part of the workpiece near the interface, higher microhardness values in the transition zone compared to the main C11000 copper mass can be caused by several factors: steel inclusions were located in the measurement (indentation) area; solid solution hardening of copper with iron occurred; and there were finer grain sizes in the transition zone compared to the main copper part of the workpiece.

The increased concentration of Fe with Cu dilution in this region explains the presence of small globular inclusions due to its increased tendency to partition. This phenomenon, with the intrusion of the underlying layers, including the substrate, has also been observed in the previous works of various additive products consisting of austenitic stainless steels, nickel-based heat resistant alloys and copper and bronze alloys [38,41,42].

### 3.3. Microstructure and Texture Features of Large-Sized C11000 Blocks

**Figure 11:**
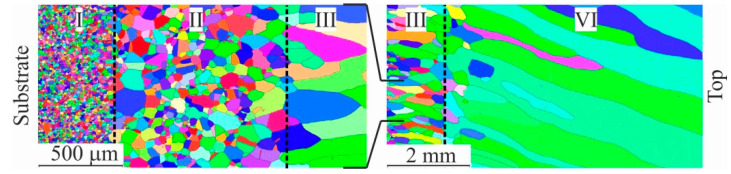
IPF maps in the Z direction of the C-type copper block. The following regions are distinguished—I: small equiaxed grains, II: large equiaxed grains, III: medium elongated grains and IV: large elongated grains.

The barrier effect (the effect of the grain size, which is described by the Hall–Petch relation) affects the copper part of the sample. According to the microstructure shown in Figure 11, after the transition zone with fine grain size is formed, directional solidification occurs in the copper part of the sample. It is accompanied by the growth of columnar grains that extend in the sample’s direction of growth. This is in accordance with the crystallization patterns of many materials that use AM, and can cause the anisotropy of mechanical properties in the samples made by the method of wire-feed 3D printing [25]. Since the growth process was continuous, one would expect the homogeneous growth of large grains throughout the entire height of the wall. Nevertheless, microstructural analysis reveals quite extended areas with equiaxed copper grains. Such features of crystallization may be caused by non-uniform heat removal from the workpiece during additive growth, changes in chemical composition and the violation of the scanning strategy. Electron backscatter diffraction (EBSD) analysis was performed to determine the direction of texturing. In the inverse pole figure (IPF) maps, several regions can be clearly distinguished: small equiaxed grains (I), large equiaxed grains (II), medium elongated grains (III), and large elongated grains (IV).

**Figure 12:**
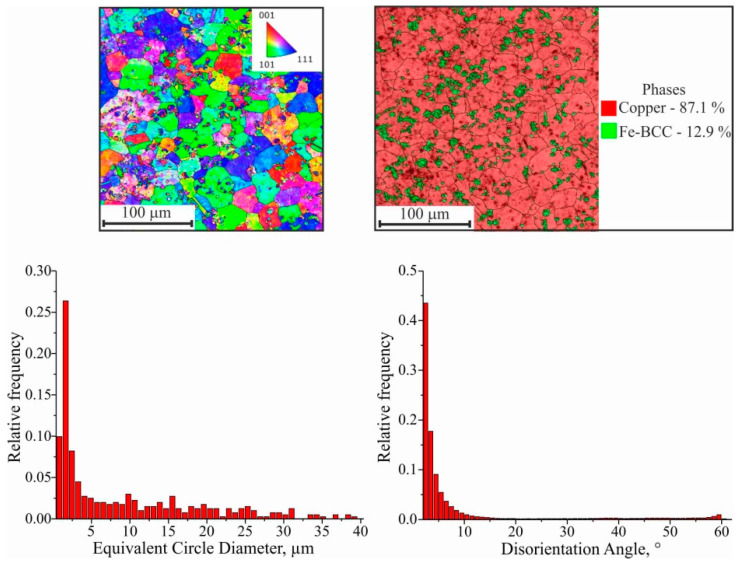
EBSD map fragment; grain size; disorientation; scattered and contoured IPFs; phase map (Cu: red color, Fe: green color).

In addition, the transition boundary from the substrate to the first layers, in which a supersaturated solid solution is formed, is considered separately (Figure 12). The deposition of the first layers of C11000 copper on AISI 304 stainless steel substrate results in the growth of some grains at the expense of neighboring ones through the migration of high-angle boundaries; i.e., primary recrystallization takes place. The formation of crystallization centers and the growth of new equilibrium grains with undistorted crystal lattice takes place. New grains arise at the boundaries of old grains where the lattice was most distorted. The number of new grains gradually increases, and no old, deformed grains remain in the structure. The driving force of primary recrystallization is the energy stored in the metal. The system tends to move to a steady state with an undistorted crystal lattice. In this case, grain boundaries migrate, and an equilibrium structure with minimal surface energy and grains of equal size and shape is formed. The impurity atoms and second-phase inclusions (in the transition zone) are factors that promote the formation of a large number of recrystallization nuclei, but which prevent the migration of grain boundaries. Another factor is the rapid heat dissipation. Since the fine-grain structure is formed close to the substrate, there is rapid heat dissipation during 3D printing when the distance from the water-cooled table is insignificant. In this case, there is a high fraction of low-angle boundaries due to crystallization processes, and a small fraction of twin boundaries are observed (Figure 12). The average size of the equiaxed grains in this range is 8.94 ± 0.04 μm.

**Figure 13:**
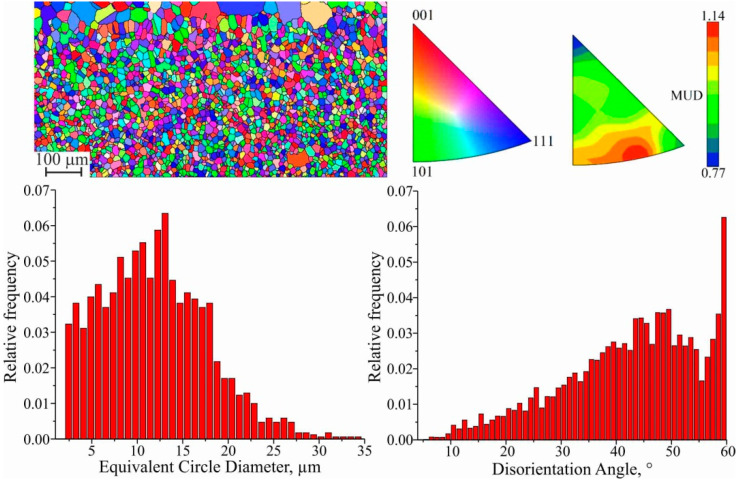
EBSD map fragment; grain size; disorientation; scattered and contoured IPFs for I area.

Since the driving force of primary recrystallization decreases as it progresses, grain growth stops when a certain value is reached. In this case, as shown by scanning electron microscopy analysis, the presence of impurity atoms with an average size of 2.5 μm in the composition of stainless steel decreases. Consequently, this factor no longer interferes with grain migration. Thus, the deposition of the first layers of C11000 copper on AISI 304 stainless steel substrate forms rounded grains with a high proportion of twin boundaries, and the proportion of small-angle boundaries decreases to a low value: a change in the angle from 10 to 40–50° increases the mobility by an order of three to five times. Since ATs are characterized by a fast printing process and the mobility of small-angle boundaries is low, the change in the volume fraction of boundaries is associated with a high temperature. Due to the large difference in the interval of angles and misorientation axes between the recrystallized grains, the mobility of the boundaries increases. This leads to an increase in the average grain size, which is d_I_ = (11.91 ± 0.03) μm (Figure 13). At the same time, the observed grains are chaotically disoriented along all three axes, and the multiple of uniform density (MUD) function at local maxima differs from the minimum only slightly. 

**Figure 14:**
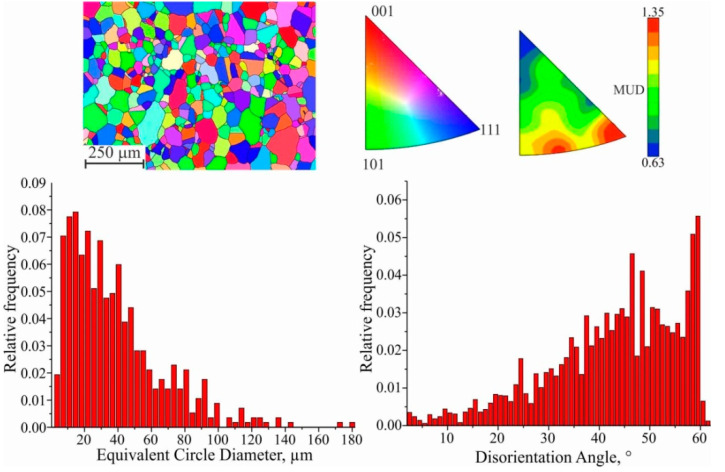
EBSD map fragment; grain size; disorientation; scattered and contoured IPFs for II area.

A remarkable feature of the microstructure of many recrystallized metals and alloys with HCC is the presence of a large number of annealing (recrystallization) twins. The twin structure is equivalent to a packing defect. The coherent surfaces of the twins turn out to be little mobile when heated, but the incoherent surfaces often migrate, which can lead to the disappearance of small twins as a result of the development of this process. Since the latter always exist in the deformed matrix, a favorable situation for twin formation is created when the growing grain meets a packing defect. However, there are a number of other features of the grain growth process that can determine the formation of twins. Further, larger equiaxed grains, with an average size of d_II_ = (27.76 ± 0.04) μm, are observed in the process of collective recrystallization (Figure 14). With small grains, the interface is large, so there is a large surface energy margin. As the grains enlarge, the total extent of the boundaries decreases and the system moves to a more equilibrium state. The ratio of large-angle, small-angle and twin grain boundaries remains the same.

**Figure 15:**
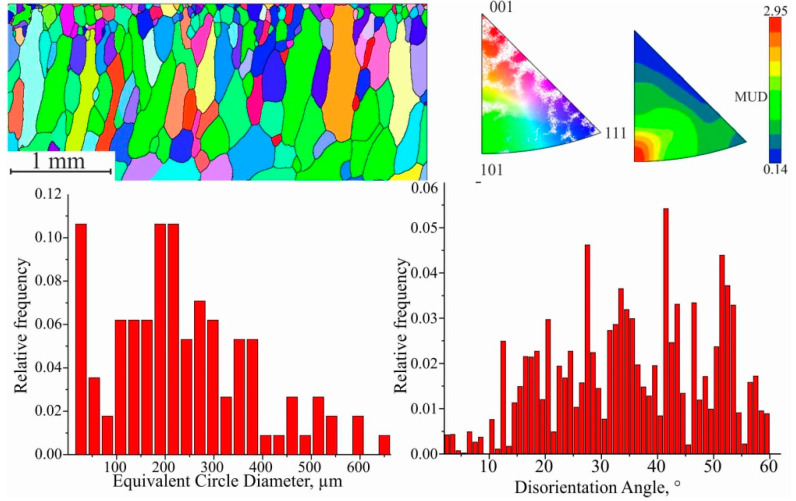
EBSD map fragment; grain size; disorientation; scattered and contoured IPFs for III area.

The formation of large grains is possible both at the expense of neighboring nuclei, where coalescence is observed, and of gradual growth. Additionally, it should be noted that the content of iron and alloying elements in the steel substrate decreases, which reduces the obstacle to the migration of grain boundaries. The predominance of chaotic grain orientation remains, but it is worth noting the formation of a predominant orientation along <101> and <111>, as evidenced by the distribution of the MUD function (Figure 15).

**Figure 16:**
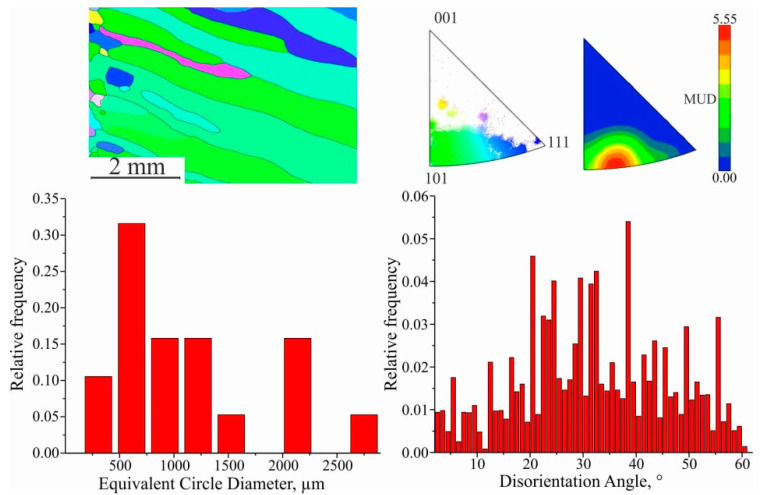
EBSD map fragment; grain size; disorientation; scattered and contoured IPFs for IV area.

The size of the grains in this region is very close to each other, but the spatial distribution of the places where the new grains emerge is different. Consequently, for orientation <101>, the distance traveled by the boundary to their collision is greater. After collective recrystallization, secondary recrystallization takes place. In this case favorably oriented grains grow, thus forming a crystallographic texture in the material. Grains of this range are characterized by an elongated shape, with an average size d_III_ = (338.54 ± 13.83) μm. The fraction of low-angle boundaries is insignificant, and twin boundaries are practically absent. Based on the IPF analysis, it is clear that the fraction of preferential orientation increases significantly, and the MUD function value differs by a factor of 25. The direction of recrystallization does not coincide with the vertical direction of the cladding due to the temperature gradient during printing. After the completion of secondary recrystallization, abnormally large, elongated grains are formed, with average length and width values of d_IV_ = (1086.45 ± 57.13) μm at the beginning of formation; in the finished workpiece, they are up to 3.5 mm and 0.3 mm in length and thickness, respectively (Figure 16). The absence of twin boundaries is characteristic.

**Figure 17:**
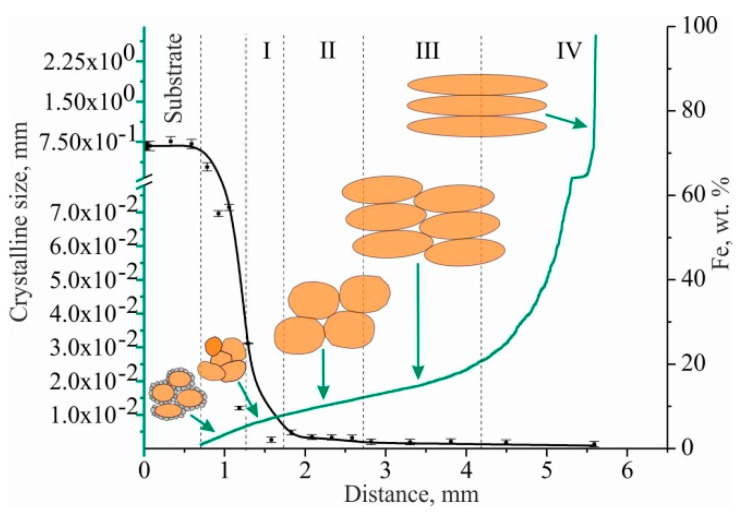
Schematic of the texture formation of C-type copper block based on the dependence of grain size on the Fe content with the distance from the substrate. Green line is crystalline size, black line is content of Fe.

Grains in this range are characterized by a preferential orientation close to <101> (range between 414 and 134). The temperature gradient plays a key role in the morphology and size of the structure and determines the crystallization mode. During the deposition of the first layers of C11000 copper on stainless steel substrate AISI 304, rapid heat dissipation occurs, which, together with the alloying of copper with austenitic steel components (primarily Fe) and the formation of inclusions of the second phase, leads to the formation of a fine-grained structure. As the blocks grows, the influence of these factors decreases and directional grain growth occurs. Directed grain growth is facilitated by the slow heat dissipation and temperature gradient formed during additive blocks growth (Figure 17).

Region II represents the orientations of those grains that can have moving boundaries, with all or most of the texture components of <101> and <111>. In region III, grains with texture orientation <101> prevent the formation of grains with texture orientation <111>. The choice of structure orientation is related to several factors. The relative volume of the texture component is related to the number of these grains, that is, the rate of their formation. In area IV, the predominant texture orientation is <101>. In this case, the growth of grains of this orientation has a minimum energy of growth.

### 3.4. Mechanical Characteristics of Large-Sized C11000 Blocks

**Figure 18:**
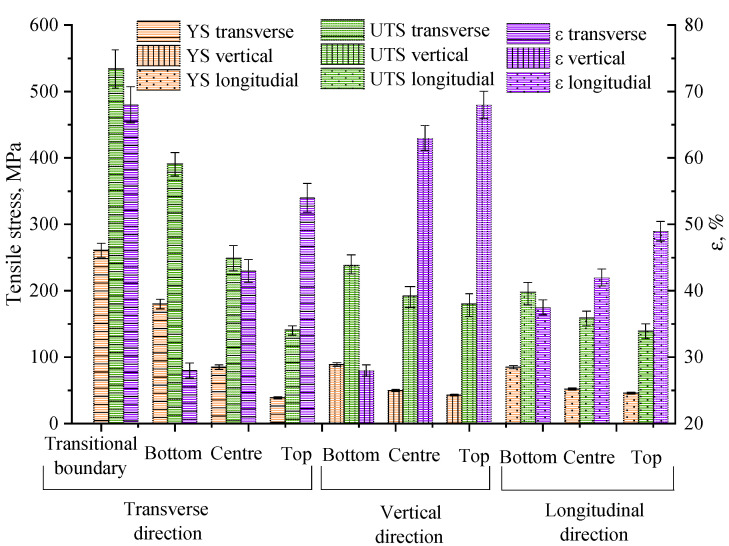
Histograms of the distribution of mechanical property characteristics of C-type copper block samples cut in transverse, vertical and longitudinal directions relative to the growing direction.

In region IV, the predominant texture orientation is 101. In this case, the growth of grains of this orientation has minimal growth energy. Since the additive growth in the copper part of the blocks forms a significantly heterogeneous grain structure, its mechanical properties may differ markedly from the properties of cast copper, and it may have anisotropy. The conducted studies of mechanical properties of samples (Figure 18), cut according to the scheme in Figure 2 from C-type blocks, show that there is a significant dependence on the material properties of copper blocks for the height of the samples from the steel substrate. In the specimens closest (transitional boundary and bottom) to the substrate, as shown earlier, there is a mixing of the steel into the additively clad copper. As a result, the copper structure is hardened by steel particles and the tensile strength σ_UTS_^trans^ reaches 534 ± 10 MPa at 2–4 mm (transitional boundary) from the substrate and 394 ± 10 MPa at 10–12 mm (bottom) for the transverse samples cut in the scanning direction 2 along the OY axis.

Further, at a distance of 18–20 mm from the substrate, there is an increase in ductility and a decrease in the tensile strength σ_UTS_^trans^ to 249 ± 10 MPa. Since the introduction of steel particles no longer leads to an increase in strength, there is a hardening due to the refinement of the grain structure of the copper near the substrate. The strength of σ_UTS_^trans^ in further samples decreases gradually (to 145 ± 10 MPa) in the upper part of the blocks, with quite similar plasticity values. This situation is due to the fact that the block structure above 20–25 mm stabilizes, and becomes a set of large grains oriented almost parallel to the direction of heat removal. A large crystal structure, defect-free, with directional growth, has high parameters of plasticity, thermal and electrical conductivity, but has lower values of ultimate strength compared to the fine-grained structure formed near the substrate. At the same time, the tensile strength of 120 MPa or more is characteristic of cast copper [18]. The mechanical test characteristics for vertical specimens cut in the build direct direction along the OZ axis differ by up to a factor of two. The mechanical test characteristics for longitudinal specimens cut in the scanning direction 1 along the OX axis are close to the values for the vertical specimens. Hence, when tensile stresses are applied in a perpendicular direction to the direction of the elongated grains in specimens with a non-uniform structure, the plastic deformation of the specimens is determined by the beginning of a slip dislocation motion in both non-uniform and uniform grains almost simultaneously. The plastic deformation of specimens whose tensile axis is parallel to the elongated grains also develops in both types of grains, because the stresses of the beginning of plastic deformation for them are close, despite the apparent difference in grain size. The effective dislocation path length in elongated grains is not significantly longer than that in equiaxial grains that are close to the substrate. The observed difference in the deformation of the experimentally obtained data presented in the Figure 18 is probably due to the influence of interfaces (grain boundaries) on the patterns of macroscopic flow of the additively grown copper crystals and requires an independent study. Thus, in the material of the studied C-type blocks, there is a heterogeneity to the mechanical properties associated with the formed structure, leading to a smooth increase in plasticity and a decrease in the strength of the material when moving away from the substrate.

## 4. Conclusions

The paper presented the production of large-sized blocks of C11000 copper on stainless steel substrates using electron beam wire-feed AM using separate perimeter formation and separate filling of the inner volume, which avoids disturbances in the macrogeometry of the samples in the printing process. It was found that the strategy of sample scanning during printing determines the formation of the structure at the macro level, and the distribution of large grains in the horizontal and vertical section of the sample. Separate filling of the perimeter does not lead to separate structure formation in the central and peripheral parts of the sample due to the smooth thermal gradient within the layer during printing. With the deposition of the first layers of C11000 copper on stainless steel substrate AISI 304, a rapid heat dissipation occurs that, together with the alloying of copper with austenitic steel components (first of all with Fe) and the formation of inclusions of the second phase, leads to the formation of a fine-grained structure d_I_ = (11.91 ± 0.03) μm. As the blocks grows, the influence of these factors decreases and directional grain growth up to 3.5 mm occurs close to the <101> orientation. Directed grain growth is facilitated by the slow heat dissipation and temperature gradient formed in the process of the additive growth of the blocks. It was established that the mechanical properties of the samples depend on the cutout area and are significantly higher in the area near the substrate (394 ± 10 MPa) at 10–12 mm (bottom) for transverse samples than the 145 ± 10 MPa recorded for the upper part of the blocks. The strength values when tested in both the horizontal and vertical directions are at the level of cast copper.

## Figures and Tables

**Table 1:** Chemical composition of the substrate and filaments used in wire-feed electron beam additive manufacturing (wt. %).

Material		Fe	Cu	Ni	Cr	Mn	Si	C
Substrate	AISI 304	Bal.	to 0.3	9–11	17–19	to 0.2	to 0.8	to 0.8
Filaments	C11000	to 0.005	Bal.	to 0.002	–	–	–	–

## Data Availability

Data sharing is not applicable to this article.
